# Burnout: exploring the differences between U.S. and international medical graduates

**DOI:** 10.1186/s12909-022-03135-x

**Published:** 2022-01-29

**Authors:** Joan E. St. Onge, Heidi Allespach, Yvonne Diaz, Alexandria Poitier, Leonardo Tamariz, Charles Paidas, Ana Palacio

**Affiliations:** 1 Department of Medicine, University of Miami Leonard M. Miller School of Medicine, 1600 NW 10th Avenue, Suite 1124, Miami, Florida 33136 USA; 2 Department of Community and Family Medicine, University of Miami Leonard M. Miller School of Medicine, Miami, Florida USA; 3 Graduate Medical Education, Jackson Memorial Hospital/Jackson Health System, Miami, Florida USA; 4 Department of Family Medicine, Morehouse University School of Medicine, Atlanta, Georgia USA; 5 Nemours A.I. duPont Hospital for Children, Wilmington, Delaware USA; 6 Department of Surgery, Sidney Kimmel Medical College, Thomas Jefferson University, Philadelphia, Pennsylvania USA; 7 Department of Public Health Sciences, Leonard M. Miller School of Medicine, Miami, Florida USA

**Keywords:** Physician burnout, Well-being, Curriculum, Medical education

## Abstract

**Background:**

International medical graduates (IMGs) have less burnout than U. S. medical school graduates (USMGs) during residency training. This study evaluates possible correlates of differences in burnout rates between USMGs and IMGs.

**Methods:**

We surveyed 375 first-year residents at orientation in June/July 2017. We assessed burnout using the Copenhagen Burnout Inventory (CBI) and used validated scales to measure stress, quality of life (QoL), mastery, and spirituality. We collected data on gender, place of graduation, language fluency, and specialty. We compared CBI scores between USMGs and IMGs, performed a multivariate linear regression analysis of relationships between covariates and CBI subscales, and logistic regression analysis for our categorical definition of burnout.

**Results:**

Two hundred twenty-two residents responded for a response rate of 59%. Personal, work or patient- related burnout was common among residents, particularly among USMGs. The most common form of burnout was work-related. Forty nine percent of USMGs have work burnout compared to 26% of IMGs (*p* < 0.01). In multivariate analysis, being an IMG reduced odds of work-related and of total burnout by 50% (OR 0.5 C.I 0.25-0.99). Perceived mastery was associated with reductions in all subscales of burnout (*p* < 0.05). Stress and low QoL related to personal and work burnout scores (*p* < 0.01).

**Conclusion:**

Work-related burnout is more common among USMGs than in IMGs. Although mastery, QoL and stress were correlates of burnout among all residents, these factors did not explain the difference. Future studies should evaluate the role of medical school structure and curriculum on differences in burnout rates between the two groups.

## Background

Burnout, a triad of emotional exhaustion, cynicism, and reduced personal accomplishment [1] affects up to 74% of physicians [2]. Medical students, residents and practicing physicians have higher rates of burnout than aged matched controls [3]. Burnout has been associated with a number of personal and professional consequences which negatively affect physician quality of life, productivity and the quality of care patients receive [4–12].

The literature has illuminated several individual-level drivers of burnout. For example, residents with greater perceived stress are more likely to report symptoms of burnout [13–16] and medical students and physicians with a lower sense of control over their lives and schedules exhibit a higher incidence of burnout [14, 17–19]. Protective factors have also been identified, and these include work-life balance [20], aerobic exercise [21], and spiritual involvement and beliefs [22–25]. These protective factors are enhanced by the use of effective coping strategies, as well as by perceptions of personal accomplishment [26, 27].

Two large cohort studies found that international medical graduates (IMGs) have lower rates of burnout than graduates of US medical schools (USMGs), and IMGs report higher ratings of quality of life (QoL), self-esteem and person growth scores [26–28]. However, these studies had limited data on personal attributes which could explain the variance between these groups. It has been demonstrated that learning styles are more self-directed in IMGs, which may be a protective factor [29]. Lower levels of debt in IMGs may also contribute to less burnout among IMGs [26]. Understanding differences and similarities between USMGs and IMGs may help identify and mitigate factors implicated in the development of burnout. The aim of this study was to evaluate both the prevalence of burnout, and the contribution of personal attributes to burnout among USMGs and IMGs at the onset of their first year of post-graduate training.

## Methods

The University of Miami Miller School of Medicine Institutional Review Board approved this research protocol. We included all incoming interns training in 21 specialties at University of Miami/Jackson Memorial Hospital (UM/JMH) and the University of South Florida Morsani College of Medicine (USF).

We distributed the 51-item survey during orientation between June 20 and July 1, 2017. The survey assessed burnout and covariates, including perceived stress, mastery, depression, spirituality, QoL and current use of wellness techniques.

We used the Copenhagen Burnout Inventory (CBI) to measure burnout due to its ability to capture revealing factors specific to burnout. The CBI, was developed by Kristensen and colleagues for use in the PUMA Study (Danish acronym for Project on Burnout, Motivation and Job Satisfaction) to investigate increasing rates of long term sick leave and early retirement in union workers [30]. They found that scores on the three measures of the CBI have been shown to predict sleep problems, use of pain killers, absence from work and intention to quit [30]. In addition, the CBI may be preferable for an international cohort, as the often-used Maslach Burnout Inventory was found to be problematic for Danish workers in the PUMA plot study, who found some of the questions were not applicable to their world view and belief system [30]. In spite of a recent uptake on the use of the CBI for the evaluation of burnout, very few studies have utilized it to study burnout in residents [31, 32] and none have used CBI to compare burnout between USMGs and IMGs.

The CBI is a 19-item questionnaire with three sub-scales that measure personal burnout, work-related burnout and client-related burnout. In our survey, we used the term, “*patients*” instead of “*clients.”* The three sub-scales of CBI use a 5-point Likert scale associated to values ranging from 0 to 100. The results of the three subscales are totaled separately and averaged to determine a total burnout score. Higher scores correlate to higher levels of burnout. All three scales have very high internal reliability, and small non-response rates. In the PUMA study, the scales differentiated well between occupational groups in the human service sector, and identified the expected pattern of correlations with other measures of fatigue and psychological well-being [30]. For categorical analysis, we used the average burnout scores reported in the PUMA study for chief physicians as a cut point for burnout [30].

The Pearlin Mastery Scale, a validated 7-item measures the extent to which “an individual feels their life chances are under their own control in contrast to being fatalistically ruled.” Higher scores indicate higher levels of mastery [33, 34]. It is important to note that “mastery” in this context does not refer to the competency to perform, but to perception of control over one’s life.

The Perceived Stress Scale 4, a validated four-item scale measures the degree to which one views life situations as stressful. Higher scores correlate to more perceived stress [35].

The Patient Health Questionnaire-2 (PHQ-2) is a 2-item screen for depression. Higher scores indicate a higher probability of a major depressive disorder [36, 37].

We measured spirituality using a one-question True/False item: **“***My spiritual beliefs are very important in helping me cope with stressors*.” [38]

To measure QoL, we used a one-item assessment “*How satisfied are you with your quality of life at this time*?” [39]

We assessed engagement in stress management strategies which have been found to be effective in the literature: cognitive-restructuring, mindfulness, diaphragmatic breathing, brief relaxation and daily self-care [40].

### Statistical analysis

We evaluated the distribution of the continuous variables using measures of central tendency and skewedness. We compared baseline characteristics by place of graduation (USMGs and IMGs) using chi square and t-test, and used t-test to compare average scores of personal, work and client-related burnout between USMGs and IMGs.

We used chi square to compare the proportion of USMGs and IMGs with personal, work, patient burnout scores higher than the averages reported in the PUMA study (categorical burnout definition). We utilized a similar strategy to compare the proportion who had scores higher than the PUMA study averages in all three subscales combined.

We conducted a multivariate linear regression analysis of relationships between all covariates and each of the three continuous subscales. We utilized logistic regression analysis for the categorical definition of burnout. The categorical outcome was having scores higher than the average reported in PUMA across the three subscales simultaneously. We report the odds ratio and corresponding 95% confidence interval (CI) for all models. Models included all covariates described above.

The fitness of the data was assessed using the deviance ratio. Analyses were performed using STATA version (College Station, Texas), and all significance tests were two-tailed.

## Results

Of 375 eligible residents (200 UM/JMH and 175 at USF) at both institutions, 222 completed the questionnaire for a response rate of 59%. Baseline characteristics of USMGs and IMGs were similar. (Table 1). Both groups had similar high QoL and mastery scores, but also similar moderate to high-perceived stress. IMGs were significantly more likely to report their spiritual beliefs helped them cope with stress and were more likely to report a primary language other than English.

**Table 1:** Baseline characteristics

Characteristic	Overall	Graduate outside of the US	Graduated in the US	*p*-value
Number	222	43	179	
Female, %	49	44	49	0.60
Non-surgical program, %	65	63	64	0.88
English is the primary language, %	65	19	77	**< 0.01**
Mastery score, mean +/−SD	23.7+/− 2.38	23.6+/− 2.4	24.0+/− 2.0	0.38
Stress score, mean +/−SD	8.6+/−1.6	8.7+/− 1.8	8.5+/− 1.6	0.38
Quality of life, mean +/−SD	5.32+/− 0.86	5.45+/− 0.88	5.28+/− 0.86	0.27
Spiritual belief, %	58	86	51	**< 0.01**
Depression, %	2	2	1	0.52
Any wellness technique, %	68	79	66	0.09
Cognitive restructuring, %	45	51	43	0.33
Mindfulness, %	38	37	38	0.92
Diaphragmatic breathing, %	21	16	22	0.38
Brief relaxation imagery, %	26	33	19	0.06
Cognitive restructuring, mean +/SD	1.8+/−0.94	1.37+/−1.09	1.22+/−1.16	0.46

The mean +/− SD for the three burnout subscales were 30.07 +/− 13.6 for personal burnout, 38 +/− 13 for work burnout and 18.8 +/− 14.6 for patient-related burnout.

**Fig. 1:**
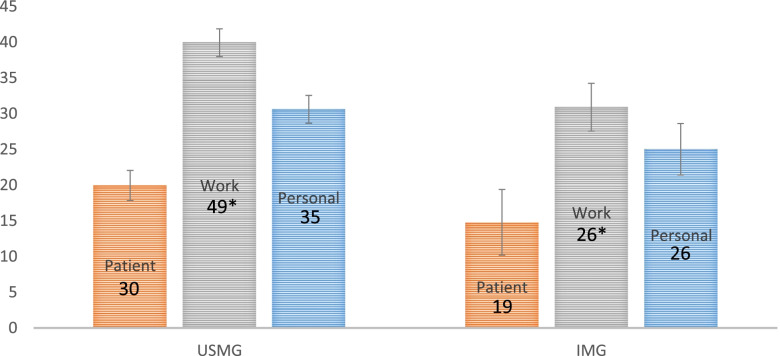
USMG vs IMG CBI Burnout Scores. USMG vs IMG CBI Burnout Scores in Patient, Work, and Personal Domains *p = <.01

We found the distribution of burnout scores differed according to place of graduation. (Fig. 1).

The largest difference between USMGs and IMGs was in work-related burnout (39.9 +/− 12.9 versus 30.9 +/− 10.5 respectively). However, personal burnout and patient-related burnout scores were also approximately 5 percentage points lower among IMGs. The creators of the scale described a 5% difference as the cut point for a significant difference in burnout [30].

### Burnout as a categorical variable

Among USMGs, one in two residents scored higher than the PUMA average for work burnout and one in three residents scored higher for personal or patient burnout. In contrast, among IMGs, one in four residents scored higher than PUMA averages for personal or work burnout and one in five for patient burnout. Interestingly, we found that 12% of USMG graduates had higher scores than PUMA for the three subscales simultaneously, compared to only 5% of IMGs. (Table 2).

**Table 2:** Prevalence of Burnout as categorical variable by place of graduation

	Entire group (*n* = 222)	US graduates (*n* = 179)	Foreign graduates (*n* = 43)	*p* value
Personal burnout,%	34	35	26	0.23
Work burnout,%	44	49	26	**< 0.01**
Patient burnout,%	27	30	19	0.11
Burnout in 3 components, %	11	12	5	0.04

### Correlates of personal, work and patient burnout

Our linear regression models found that associations between individual-level covariates and burnout varied across each of the three subscales: We found that higher mastery, or having more control over life’s events, lower perceived stress and higher QoL were associated with less personal and work-related burnout (*p* < 0.05). The strongest correlate of work burnout was place of medical school graduation. International medical school graduation was associated with a reduction of almost 10 points in the work burnout score (C.I. -15.05487-4.559158). Being female reduced the patient-related burnout score by 4.5 points (C.I. -8.49657-.4862486) and each point increase in mastery reduced the patient burnout by 1.3 points (C.I. -2.135038-.425182). Specialty, use of wellness techniques, and spirituality did not correlate with any burnout score. (Table 3).

**Table 3:** Beta-coefficients for correlates of three burnout subscales

Correlates	Personal burnout	Work burnout	Patient burnout
UM/JMH vs USF	−.56	.89	−1.39
Women vs men	1.45	.35	**−4.49**^**3**^
Did not graduate in US	−3.64	**−9.80**^**1**^	−2.67
Non-surgical vs surgical	−2.78	− 2.21	1.22
Fluent in English	4.20	−3.24	3.87
Mastery	**−1.20**^**1**^	**−1.24**^**1**^	**−1.28**^**2**^
Stress	**1.41**^**2**^	**1.05**^**3**^	.90
Total wellness techniques	1.05	.72	−.65
Depression	.69	−6.30	.99
Quality of life	**−3.33**^**1**^	**−3.65**^**1**^	−2.02
Spiritual	−.77	−1.87885	−1.65

^1^ *p* _< 0.001_

2 *p* < 0.01

3 *p* < 0.05

### Correlates of combined burnout in all three subscales

The logistic regression model found that IMGs (*p = 0.04*) and those with higher perception of mastery (*p < 0.01*) were significantly less likely to be burned out on the three burnout subscales simultaneously. Residents who indicated English as their primary language were almost twice as likely to report burnout compared to residents who indicated another primary language (*p = 0.02*) when adjusting for all other variables. (Table 4).

**Table 4:** Multivariate predictors of having burnout in 3 subscales

Predictor	OR (95% CI)	*p*-value
UM/JMH vs USF	1.15(0.66-1.98)	0.60
Women vs men	1.30(0.77-2.19)	0.32
Non-surgical vs surgical	0.93(0.53-1.61)	0.80
Did not graduate in US	0.50(0.25-0.99)	**0.04**
Mastery	0.80 (0.71-0.90)	**< 0.01**
Stress	1.12(0.96-1.32)	0.13
Fluent in English	1.84(1.07-3.16)	**0.02**
Spiritual	0.55(0.23-1.31)	0.18
Quality of life	0.71(0.41-10)	0.11
Total wellness techniques	0.96(0.68-1.35)	0.83
Depression	0.41(0.06-2.5)	0.37

## Discussion

Our study found higher prevalence of burnout among USMGs than in IMGs, consistent with previous research [19, 26, 27]. Importantly, our study contributes information on burnout correlates that has been lacking. We found that work-related burnout drives the difference between USMG and IMGs at the start of residency. However, the personal factors we evaluated did not explain the burnout difference. Perceived mastery, stress and QoL correlated with burnout among all residents.

Prior studies reported that English-speaking residents and those raised in the US or Canada were more likely to have higher rates of emotional exhaustion and depersonalization [41]. Low QoL has been correlated with symptoms of burnout [26]. However, studies evaluating other factors which account for the differences in burnout between USMGs and IMGs are limited. Increased resilience by virtue of successfully integrating into a US residency program may lead to a greater sense of mastery or lower perceived stress in IMGs when compared to USMGs. In our study, we did not see a difference in these covariates between these two groups. This is consistent with a 2009 study of internal medicine residents that showed no difference in perceived stress between IMGs and USMG during their residency [28]. Our study found that IMGs relied more on spirituality to cope with stress. While research has shown spirituality to have a protective effect against burnout in other groups [22–24, 42] spirituality was not a significant predictor of burnout in our study. A different prevalence of depression may also be a factor; however, in our cohort, the rate was low in both groups. The relationship between depression and burnout remains unclear [43].

The lack of differences in personal attributes between USMGs and IMGs suggests that aspects of the medical school experience may play a predominant role in burnout development. At the start of medical school, US medical students have shown lower rates of burnout and depression and higher QoL than aged-matched controls, indicating that burnout develops during medical school [44]. Therefore, differences in medical school curricula and experiences between USMGs and IMGs may help explain the difference in burnout. International medical graduates typically enter school immediately after high school, and complete 2-3 years of preclinical study followed by 3-4 years of clinical study leading to a shorter premedical period and earlier clinical exposure [45]. Chen theorized that the perception that the United States Medical Licensing Exam (USMLE) Step 1 scores dictate a student’s “worthiness” for higher achievement has contributed to increasing burnout in physicians and physicians-in- training [46]. While Step 1 scores may affect the future career of IMGs, these physicians usually take the test after medical school graduation. The recent decision by the National Board of Medical Examiners to change the USMLE Step 1 Exam to pass/fail grading [47] may help to mitigate this concern and elucidate the contribution of this high stakes exam to burnout.

It is important to note that higher perceived mastery, or a greater perception of having control over life’s events, was associated with lower burnout scores in both cohorts. In addition, our finding that stress and QoL correlate to personal burnout suggests that contextual factors in the lives of USMGs and IMGs during medical school play a role in their coping abilities. Future studies should evaluate predictors of stress, QoL and perceived mastery among medical students. Our study had several limitations. The cross-sectional nature of our study prevented us from evaluating causality. While we used many validated scales, two of our measures were adapted to our cohort. We used a single item to measure QoL, which may not accurately reflect the QoL of our residents and only one question to ascertain the role of spiritual beliefs in coping with stress. To maintain anonymity of residents, we limited the specialty choice to “surgical” and “non-surgical” which did not allow us to analyze whether a specific specialty choice is a factor in burnout in our cohort. Residents reported their practice of wellness techniques, but we did not ascertain the quality or fidelity of their practice. The amount of debt incurred has been found to affect burnout rates in residents [26], but we did not measure debt in our study. We only included first year residents limiting our sample size and our ability to compare burnout and its predictors at different levels of training. In addition, comparing intern’s responses with more senior residents or fellows would have further elucidated the relationship between self-perceived mastery (control over the events in one’s life) and burnout. Importantly, it is possible that response bias contributed to the results of the study. IMGs may have felt pressure to answer the survey in what they perceived a more favorable manner than the USMGs. While the results of three of the subscales of the CBI found no statistical difference between USMGs and IMGs, response bias limits the interpretation of the overall results, and reinforces the need for study in more senior residents and fellows. Finally, the lack of direct measurements of health such as detailed medical histories or sleep assessments limit the clinical relevance of these findings.

The strengths of our study included its cohort: a diverse, multispecialty sample of residents from two institutions at the onset of training. Our evaluation of the role of mastery, perceived stress, QoL, spirituality and the practice of wellness techniques on burnout contributes to the literature that examines burnout rates in USMGs vs IMGs. Our findings have important implications regarding future directions of inquiry.

Finally, an additional strength is the utilization of the CBI. This scale provides a measurement of burnout that allows us to recognize the different burden of burnout in the personal, work and patient-related domains. In the PUMA study, results of the CBI in health-related workers correlated with outcomes such as a job satisfaction, sick days, sleep problems, and intention to quit work [30], increasing its relevance for evaluation of burnout in postgraduate learners, indicating clinical relevance of these findings. However, future studies using the CBI and other health assessments in USMGs and IMGs during training and beyond are needed to ascertain the true predictive clinical value of the results. The CBI’s use and validity in studying an international cohort are important as we look beyond personal characteristics to study the institutional correlates of burnout.

## Conclusion

At the onset of post-graduate training USMGs have a higher prevalence of work-related burnout than IMGs, a difference not explained by personal attributes. However, the use of the CBI identified that work-related burnout appeared to drive the difference between USMG and IMGs at the start of residency. Perceived control over one’s life, stress and QoL correlated with burnout among all residents. IMGs relied more on spirituality to cope with stress, but spirituality was not a significant predictor of burnout in multivariate analysis.

Further research should evaluate the role of medical education structure, curriculum and environmental factors on burnout and impact of interventions that improve mastery and reduce stress in our learners. Continuing study on the differences between international and US medical education and effects on burnout and other health issues is an important component of future study.

## Data Availability

The datasets generated and/or analyzed during the current study are not publicly available due to requirements of the institutional review board, but are available from the corresponding author on reasonable request.

## Abbreviations

CBI: Copenhagen Burnout Inventory
CI: Confidence Interval
IMG: International medical graduate
PHQ2: Patient Health Questionnaire 2
PUMA: Danish acronym, translated in English to “Project on Burnout, Motivation and Job Satisfaction”
QoL: Quality of life
UM/JMH: University of Miami/Jackson Memorial Hospital
US: United States
USF: University of South Florida Morsani School of Medicine
USMG: United States medical graduate
USMLE: United States Medical Licensing Examination
